# Metaplastic Breast Cancer: Characteristics and Survival Outcomes

**DOI:** 10.7759/cureus.28551

**Published:** 2022-08-29

**Authors:** Bicky Thapa, Salome Arobelidze, Bernadette A Clark, Jia Xuefei, Hamed Daw, Yee Chung Cheng, Mita Patel, Timothy PP Spiro, Abdo Haddad

**Affiliations:** 1 Hematology and Oncology, Medical College of Wisconsin, Milwaukee, USA; 2 Internal Medicine, Cleveland Clinic Lerner College of Medicine/Case Western Reserve University (CWRU), Cleveland, USA; 3 Hematology and Oncology, Cleveland Clinic Fairview Hospital, Cleveland, USA; 4 Quantitative Health Sciences, Cleveland Clinic, Cleveland, USA; 5 Surgery and Oncology, Mercy Regional Medical Center, Lorain, USA

**Keywords:** triple negative, prognosis, hormone receptors, radiation, mastectomy, chemotherapy, histology, ecog, metastasis, metaplastic breast cancer

## Abstract

Objectives

Metaplastic breast cancer (MBC) is a rare neoplasm accounting for <1% of all breast cancer. We evaluated the clinical characteristics and survival outcomes of MBC.

Methods

Patients diagnosed with pathologically proven MBC were reviewed from the institutional breast cancer database from 2000 to 2017.

Results

A total of 136 patients diagnosed with MBC were included in the study. The median age of the diagnosis was 60 years, and 60% of patients were stage II at diagnosis, and 22% were stage III. About two-thirds of the patients were triple-negative; 93% had nuclear grade III, and 25% had a lymphovascular invasion. Squamous differentiation (29%) was the most common histologic subtype, followed by the spindle subtype (21%). The most common distant metastases were lung (22%), followed by bone (13%). Moreover, 60% had a mastectomy, 19% had endocrine therapy, 58% had radiation, 51% received anthracycline-based chemotherapy, 26% had non-anthracycline chemotherapy, and 22% received no chemotherapy. In the entire cohort, the two-year overall survival (OS) and five-year OS were 79% and 69%, respectively, and the two-year progression-free survival (PFS) and five-year PFS were 72% and 61%, respectively. On multivariable analysis, the stage of MBC (stage III: hazard ratio (HR), 5.065 (95% confidence interval (CI), 1.02-25.27) (p=0.048)), poor functional status (Eastern Cooperative Oncology Group (ECOG) score, 2; HR, 24.736 (95% CI, 1.92-318.73) (p=0.014)), and distant metastasis to the brain (HR, 8.453 (95% CI, 1.88-38.04) (p=0.005)) and lung (HR, 42.102 (95% CI, 7.20-246.36) (p<0.001)) were significant predictors of decreased OS.

Conclusions

MBC demonstrated early disease progression and poor overall survival. The stage of MBC, decreased performance status, and metastasis to the lung and brain were independent poor prognostic factors.

## Introduction

Metaplastic breast cancer (MBC) is a rare neoplasm that accounts for less than 1% of all breast cancers, and it is characterized by histologic and molecular heterogeneity. MBC is histologically characterized by the differentiation of the neoplastic epithelium into squamous cells and/or mesenchymal-looking elements [1]. Evidence also suggests that MBC has an aggressive nature and tends to have a worse prognosis of MBC as compared to other breast cancers. This is possibly due to its rarity, tumor heterogeneity, and lack of targeted treatment [2,3]. Various clinical and immunohistochemical factors along with genetic markers have been described in the literature; however, no validated prognostic markers have been identified. To date, MBC remains a clinical challenge for physicians regarding pathogenesis, clinicopathological features, and its management [2]. Most MBCs are triple-negative; hence, they are managed in a similar way to triple-negative breast cancer (TNBC) with anthracycline, taxane, and platinum-based therapy. There is no standard therapeutic approach available for this breast cancer subtype.

In this study, we evaluated the clinical, histopathologic, and molecular characteristics across all locations of our health system. We also sought to identify potential factors attributing to the progression of the disease and survival.

## Materials and methods

Patients

The complete list of the study cohort from January 2000 to December 2017 was identified from a report generated from the institutional database. The study was approved by the Cleveland Clinic Institutional Review Board (IRB# 17-404). The database for pathologically proven MBC cases was maintained in the breast tumor registry from three centers in the Cleveland Clinic health system. The inclusion criteria for the study include age > 18 years, female gender, and all pathologically diagnosed and proven cases of MBC from the database. Patients aged <18 years and males were excluded. A total of 136 patients fulfilled the inclusion criteria and were included in the study for analysis.

The retrospective chart review was done using an electronic medical record for data abstraction. Study variables for data collection include age at the diagnosis, demographics, Eastern Cooperative Oncology Group (ECOG) performance status, histopathology, staging, hormone receptor status, lymphovascular invasion, molecular markers, treatment received (surgery, radiation therapy, chemotherapy, and hormonal therapy), date of local and distant progression, and date of death or last follow-up. The stage of MBC was documented based on the breast cancer staging system by the American Joint Committee on Cancer (AJCC).

Pathology

The histopathology of breast tissue was evaluated by an expert pathologist in breast cancer and documented in the electronic medical record. MBC was defined based on the World Health Organization (WHO) classification for breast tumors [1]. It is a unique group of invasive ductal carcinoma, which is characterized by the differentiation of tumor cells into purely epithelial or mixed epithelial and mesenchymal components. The epithelial group includes squamous, adenocarcinoma with spindle cell differentiation, and adenocarcinoma, including mucoepidermoid. Mixed epithelial and mesenchymal components are comprised of carcinoma with chondroid metaplasia, carcinoma with osseous metaplasia, and carcinosarcoma. We broadly categorized histology into four groups for analysis as follows: squamous subtype, spindle cell subtype, mixed epithelial plus mesenchymal differentiation, and other types of metaplastic carcinoma with no special type. Other histology of MBC with no special type includes ductal carcinoma in situ micropapillary pattern, heterologous type with matrix production, high-grade metaplastic carcinoma, large cells with intermingling mature plasma cells, matrix-producing carcinoma, and sarcomatoid features, and metaplastic carcinoma.

Using immunohistochemical stains at our institution, *p53*, estrogen receptor (ER), progesterone receptor (PR), and human epidermal growth factor receptor 2 (HER2) status were assessed. Variables were collected for histologic subtypes; nuclear grades; lymphovascular invasion; PR, ER, and HER2 status; and *p63* and *BRCA* mutation.

Statistical analysis

Categorical data are summarized as frequencies and percentages, whereas continuous data as medians and ranges. Overall survival (OS) and progression-free survival (PFS) were the primary outcomes. OS was calculated from the date of the diagnosis of metaplastic breast cancer to the date of death or last follow-up. PFS was calculated from the date of the diagnosis of MBC to the date of distant progression or local progression of disease or death or follow-up, whichever comes first. Time to event variable was summarized using the Kaplan-Meier method; the log-rank test and univariate cox regression model were used to estimate the association between outcomes and patient clinical and pathological characteristics, including tumor-node-metastasis (TNM) stage (I-IV), distance metastasis, hormone receptor status, histologic subtypes, type of therapy, and performance status. The variables that were selected through stepwise selection were included in the multivariable Cox model. All statistical analyses were performed using R version 3.5.0. p-value < 0.05 was considered statistically significant.

## Results

Patient characteristics

The median age at diagnosis was 60 years (27-92 years), with a median follow-up of 51.5 months. Most of the patients were white (n=101 (74%)), followed by black (n=31 (23%)). ECOG performance status was classified as 0, 1, 2, and 3 in 77 (57%), 38 (28%), 12 (9%), and nine (6%) patients, respectively. Lymphovascular invasion was demonstrated in 27 (25.7%) patients; 114 (93.4%) had nuclear grade III, five (4%) had nuclear grade II, and three (2.4%) had nuclear grade I. Eighty-two (60.3%) patients were diagnosed at stage II, 28 (20.6%) at stage I, 19 (14%) at stage III, and seven (5.1%) at stage IV. A total of 101 (74.3%) patients were triple-negative. Estrogen receptor (ER), progesterone receptor (PR), and HER2/neu expression were positive in 22 (16.2%), 12 (8.8%), and 14 (10.3%) patients, respectively; only one patient was triple-positive. On further breakdown for hormone receptor positivity, nine patients had HER2/neu expression, 10 had ER, four had both HER2 and ER, seven had both ER and PR, four had PR only, and one had all three receptors positive.

On histologic evaluation, spindle cell tumor was observed in 29 (21%) patients, and 40 (29%) patients showed squamous subtype. Fifty-five (40%) patients had mixed epithelial plus mesenchymal carcinoma, and 12 (9%) were found to be other types of metaplastic carcinoma with no specific type. Breast pathologists documented the diagnosis of MBC in the chart. Histology documentation in the pathology report was utilized to divide MBCs into different subtypes. Seven patients had *BRCA* mutations (five had *BRCA1* and two had *BRCA2*). Forty-three (31.6%) patients expressed *p63*. The most common distant metastases were observed in the lungs (n=30 (22%)), followed by the bone (n=18 (13%)) and brain (n=9 (7%)). The adrenal and spleen were rare sites of metastasis, each occurring in one patient. Fifty (40%) patients had a lumpectomy, 76 (60%) had a mastectomy, 25 (20%) received hormonal therapy, 76 (58%) received radiation, 51% received anthracycline-based chemotherapy, 26% had non-anthracycline chemotherapy, and 22% received no chemotherapy. Unfortunately, not all clinical and pathological data were available for patients included in the study during the chart review. Complete demographics, clinical, and pathological variables are outlined in Table 1.

**Table 1:** Patient characteristics AJCC, American Joint Committee on Cancer; *p63*, tumor protein *p63*; MBC, metaplastic breast cancer; ECOG, Eastern Cooperative Oncology Group; NA, data not available from the chart review; HER2, human epidermal growth factor receptor 2 #Other histology in our study includes ductal carcinoma in situ micropapillary pattern, heterologous type with matrix production, high-grade metaplastic carcinoma, large cells with intermingling mature plasma cells, matrix-producing carcinoma, and sarcomatoid features, and metaplastic carcinoma.

Variables	Level	Overall
Age at diagnosis (median (range))		60 (27, 92)
Race (%)	Black	31 (23.5)
	White	101 (76.5)
	Others	4 (2.9)
ECOG performance status (%)	0	77 (56.6)
	1	38 (27.9)
	2	12 (8.8)
	3	9 (6.6)
Lymphovascular invasion (%)	No	78 (57.3)
	Yes	27 (19.8)
	NA	31 (22.8)
*p63* (%)	Negative	93 (68.4)
	Positive	43 (31.6)
*BRCA* status (%)	*BRCA1* positive	5 (3.7)
	*BRCA2* positive	2 (1.5)
	NA	129 (94.8)
Nuclear grade (%)	I	3 (2.4)
	II	5 (4)
	III	114 (93.4)
	NA	14
AJCC stage (%)	I	28 (20.6)
	II	82 (60.3)
	III	19 (14)
	IV	7 (5.1)
Hormonal therapy (%)	No	103 (80.5)
	Yes	25 (19.5)
	NA	8
Radiation therapy (%)	No	55 (42)
	Yes	76 (58)
	NA	5
Chemotherapy type (%)	Anthracycline	70 (51.5)
	Non-anthracycline	36 (26.5)
	None	30 (22)
Surgery (%)	Mastectomy	76 (60.3)
	Lumpectomy	50 (39.7)
Histologic subtypes (%)	Spindle cell	29 (21.3)
	Squamous	40 (29.4)
	Mixed MBC/mesenchymal	55 (40.4)
	^#^Others	12 (8.8)
Triple-negative status (%)	No	35 (25.7)
	Yes	101 (74.3)
Estrogen receptor positivity (%)	No	114 (83.8)
	Yes	22 (16.2)
Progesterone receptor positivity (%)	No	124 (91.2)
	Yes	12 (8.8)
HER2 expression (%)	No	122 (89.7)
	Yes	14 (10.3)
Metastasis to the lung (%)	No	106 (77.9)
	Yes	30 (22.1)
Metastasis to the liver (%)	No	130 (95.6)
	Yes	6 (4.4)
Metastasis to the bone (%)	No	118 (86.8)
	Yes	18 (13.2)
Metastasis to the adrenal (%)	No	135 (99.3)
	Yes	1 (0.7)
Metastasis to the spleen (%)	No	135 (99.3)
	Yes	1 (0.7)
Metastasis to the brain (%)	No	127 (93.4)
	Yes	9 (6.6)

Survival analysis

At the time of analysis, a total of 44 patients had died, and 36 patients were found to have progression (local or distant). Four patients were excluded from survival analysis due to missing information. Two patients had missing dates of diagnosis, and two patients had missing dates of the last follow-up or date of death. The two-year and five-year OS rates in the entire cohort were 79% and 69%, respectively. We observed a two-year PFS rate of 72% and a five-year PFS rate of 61%.

Progression-free survival

**Figure 1:**
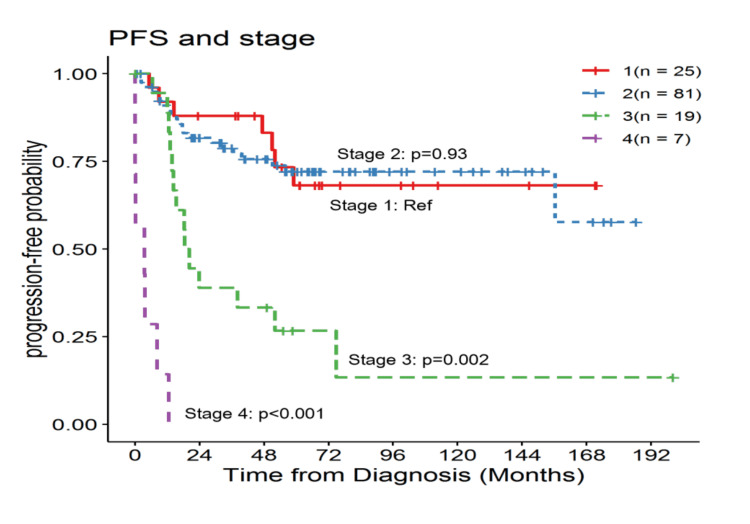
Stage-by-stage progression-free survival probability curve PFS: progression-free survival; Ref: reference

Univariable analysis for PFS was statistically significant for the stage of the MBC and higher functional status (ECOG 0 and 1 ). Lumpectomy and mastectomy were the types of surgery included as a variable for analysis. The five-year PFS rates for stages I, II, and III were 68%, 72%, and 27%, respectively (Figure 1). Stage IV had worse survival with only a one-year PFS rate of 14%. Metastases to the lung, brain, bone, and liver were found to have worse PFS. The complete univariable analysis is summarized in Table 2.

**Table 2:** Univariable analysis for progression-free survival AJCC, American Joint Committee on Cancer; MBC, metaplastic breast cancer; N, number of patients; HR, hazard ratio; CI, confidence interval; NA, not applicable; Ref, reference; ECOG, Eastern Cooperative Oncology Group; HER2, human epidermal growth factor receptor 2 ^Four patients were excluded from survival analysis due to missing information such as the date of diagnosis, last follow-up, or date of death. *Progression was treated as an event and death as a competing risk event. #Other histology in our study includes ductal carcinoma in situ micropapillary pattern, heterologous type with matrix production, high-grade metaplastic carcinoma, large cells with intermingling mature plasma cells, matrix-producing carcinoma, and sarcomatoid features, and metaplastic carcinoma.

Variable	Variable level	N^	Number of events*	One-year rate (% (range))	Two-year rate (% (range))	Five-year rate (% (range))	HR (95% CI)	p-value
Race	Black	31	13	94% (77%-98%)	71% (52%-84%)	61% (41%-75%)	Ref	
	White	97	35	86% (77%-92%)	73% (62%-81%)	60% (48%-70%)	0.93 (0.49-1.76)	0.83
Lymphovascular invasion	No	77	23	91% (81%-95%)	81% (70%-88%)	65% (51%-75%)	Ref	
	Yes	27	13	89% (69%-96%)	64% (43%-80%)	56% (34%-73%)	1.62 (0.81-3.24)	0.17
ECOG performance status	0	74	18	94% (86%-98%)	85% (75%-92%)		Ref	
	1	37	12	89% (74%-96%)	78% (61%-88%)		1.40 (0.67-2.91)	0.37
	2	12	12	58% (27%-80%)	17% (3%-41%)		12.19 (5.44-27.30)	<0.001
	3	9	7	67% (28%-88%)	22% (3%-51%)		8.46 (3.31-21.60)	<0.001
p63	Negative	89	35	87% (78%-93%)	70% (59%-78%)	59% (48%-69%)	Ref	
	Positive	43	14	88% (73%-95%)	78% (62%-88%)	64% (45%-78%)	0.92 (0.49-1.71)	0.78
AJCC stage	I	25	7	92% (72%-98%)	88% (76%-100%)	68% (51%-91%)	Ref	
	II	81	21	91% (82%-96%)	82% (73%-91%)	72% (62%-83%)	1.05 (0.45-2.48)	0.90
	III	19	14	94% (67%-99%)	39% (22%-69%)	27% (12%-59%)	4.17 (1.67-10.40)	0.002
	IV	7	7	14% (1%-46%)			44.64 (13.40-148.71)	<0.001
Hormonal therapy	No	100	36	88% (79%-93%)	74% (64%-82%)	62% (51%-72%)	Ref	
	Yes	24	9	91% (70%-98%)	74% (51%-87%)	59% (35%-76%)	1.04 (0.50-2.17)	0.91
Radiation therapy	No	53	19	81% (67%-89%)	71% (57%-82%)	63% (47%-75%)	Ref	
	Yes	74	28	93% (84%-97%)	73% (61%-82%)	60% (47%-70%)	0.99 (0.55-1.77)	0.97
Chemotherapy type	Anthracycline	68	21	93% (83%-97%)	80% (69%-88%)	70% (56%-80%)	Ref	
	Non-anthracycline	36	13	85% (68%-94%)	73% (55%-85%)	56% (35%-72%)	1.48 (0.74-2.98)	0.27
Surgery	Mastectomy	74	28	89% (79%-94%)	74% (62%-82%)	61% (48%-72%)	Ref	
	Lumpectomy	48	13	96% (84%-99%)	82% (68%-91%)	69% (52%-81%)	0.66 (0.34-1.28)	0.22
Histologic subtypes	Spindle cell	27	11	92% (73%-98%)	73% (51%-86%)	56% (33%-74%)	Ref	
	Squamous	40	19	82% (67%-91%)	65% (48%-78%)	54% (37%-68%)	1.15 (0.55-2.42)	0.71
	Mixed MBC/mesenchymal	54	16	88% (76%-95%)	76% (62%-86%)	66% (50%-78%)	0.69 (0.32-1.49)	0.34
	^#^Other	11	3	91% (51%-99%)	82% (45%-95%)	72% (35%-90%)	0.54 (0.15-1.95)	0.35
Estrogen receptor	No	111	41	88% (80%-93%)	73% (63%-80%)	61% (50%-70%)	Ref	
	Yes	21	8	86% (62%-95%)	71% (47%-86%)	61% (36%-78%)	1.00 (0.47, 2.14)	> 0.99
Progesterone receptor	No	121	46	86% (79%-91%)	71% (62%-78%)	60% (50%-69%)	Ref	
	Yes	11	3	100% (NA)	90% (47%-99%)	64% (24%-87%)	0.66 (0.20-2.11)	0.48
HER2 expression	No	119	45	87% (79%-92%)	73% (63%-80%)	59% (49%-68%)	Ref	
	Yes	13	4	92% (57%-99%)	69% (37%-87%)	69% (37%-87%)	0.76 (0.27-2.12)	0.60
Metastasis to the lung	No	102	20	95% (88%-98%)	84% (75%-90%)		Ref	
	Yes	30	29	63% (44%-78%)	33% (18%-50%)		11.41 (6.15-21.18)	<0.001
Metastasis to the bone	No	114	31	90% (83%-94%)	79% (70%-85%)		Ref	
	Yes	18	18	72% (46%-87%)	33% (14%-55%)		6.65 (3.60-12.28)	<0.001
Metastasis to the brain	No	123	40	87% (80%-92%)	76% (67%-83%)		Ref	
	Yes	9	9	89% (43%-98%)	22% (3%-51%)		5.00 (2.38-10.50)	<0.001
Metastasis to the liver	No	126	43	89% (81%-93%)	74% (65%-81%)		Ref	
	Yes	6	6	67% (19%-90%)	33% (5%-68%)		5.16 (2.15-12.36)	<0.001

In multivariable analysis, the stage of MBC (stage II: HR, 5.15 (95% CI, 1.00-26.52) (p=0.05); stage III: HR, 35.90 (95% CI, 5.64-228.59) (p<0.001)), metastasis to the lung (HR, 67.01 (95% CI, 16.65-269.63) (p<0.001)), and metastasis to the brain (HR, 12.28 (95% CI, 3.29-45.84) (p<0.001)) were independent predictors of progression (Table 3). Patients with liver metastasis were observed to have less tendency for progression with an HR of 0.14 (95% CI, 0.03-0.70) (p=0.016).

**Table 3:** Multivariable analysis for progression-free survival HR, hazard ratio; CI, confidence interval; Ref, reference *Analysis was done using a reference level of no hormonal therapy and no lung, liver, and brain metastases.

Variable	HR (95% CI)	p-value
Stage II (ref = stage I)	5.15 (1.00-26.52)	0.05
Stage III (ref = stage I)	35.90 (5.64-228.59)	<0.001
Stage IV (ref = stage I)	4,149.63 (139.71-123,249.44)	<0.001
^*^Hormonal therapy (ref = no)	0.27 (0.07-1.02)	0.054
^*^Lung metastasis (ref = no)	67.01 (16.65-269.63)	<0.001
^*^Liver metastasis (ref = no)	0.14 (0.03-0.70)	0.016
^*^Brain metastasis (ref = no)	12.28 (3.29-45.84)	<0.001

Overall survival

**Figure 2:**
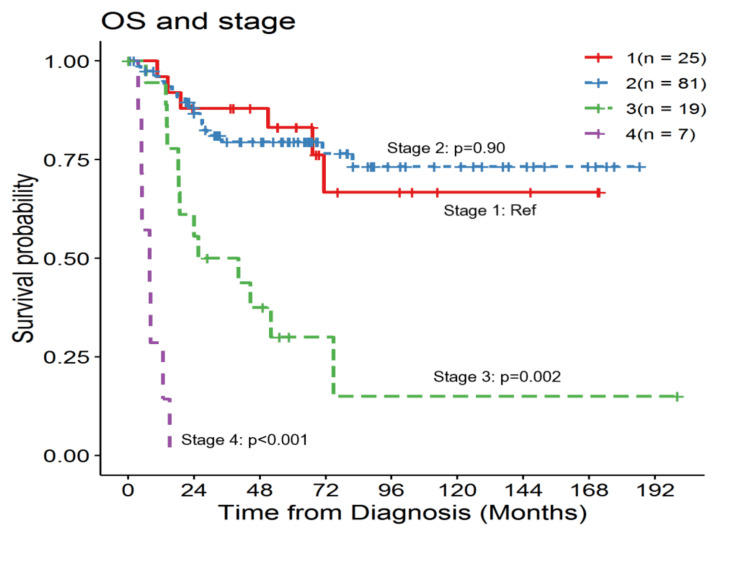
Stage-by-stage overall survival probability curve OS, overall survival; Ref: reference

The five-year OS rate at stage I was 83%. There was no difference in OS between stage I and stage II. On univariable analysis with stage I as the reference, the five-year OS rate at stage II was 79% with an HR of 0.96 (95% CI, 0.38-2.44) (p=0.93). The five-year OS at stage III was 30% with an HR of 4.53 (95% CI, 1.71-12.01) (p=0.002). For stage IV, only 29% survived at the end of one year with an HR of 43.26 (95% CI, 12.34-151.64) (p=0.001) (Figure 2). Better functional or performance status (ECOG 0 and 1) demonstrated better five-year OS, and poor functional or performance status (ECOG 3 and 4) was correlated with increased mortality. Lymphovascular invasion, *p63* positivity, hormonal therapy, radiation therapy, and histologic subtype did not show better outcomes.

Metastasis to the lung and brain at any time during follow-up was associated with poor survival. In patients with lung metastasis, the two-year and five-year OS rates were 53% and 27%, respectively, with an HR of 7.85 (95% CI, 4.18-14.72) (p<0.001). The complete analysis is summarized in Table 4.

**Table 4:** Univariable analysis for overall survival AJCC, American Joint Committee on Cancer; MBC, metaplastic breast cancer; N, number of patients; HR, hazard ratio; CI, confidence interval; NA, not applicable; Ref, reference; ECOG, Eastern Cooperative Oncology Group; HER2, human epidermal growth factor receptor 2 ^Four patients were excluded from survival analysis due to missing information such as the date of diagnosis, last follow-up, or date of death. *Progression was treated as an event and death as a competing risk event. #Other histology in our study includes ductal carcinoma in situ micropapillary pattern, heterologous type with matrix production, high-grade metaplastic carcinoma, large cells with intermingling mature plasma cells, matrix-producing carcinoma, and sarcomatoid features, and metaplastic carcinoma.

Variable	Variable level	N^^^	Number of events^*^	One-year rate (% (range))	Two-year rate (% (range))	Five-year rate (% (range))	HR (95% CI)	p-value
Race	Black	31	12	94% (77%-98%)	81% (62%-91%)	63% (43%-78%)	Ref	
	White	97	30	91% (83%-96%)	78% (68%-85%)	70% (60%-79%)	0.88 (0.45-1.71)	0.70
Lymphovascular invasion	No	77	20	95% (86%-98%)	86% (76%-92%)	75% (63%-84%)	Ref	
	Yes	27	10	96% (76%-99%)	72% (50%-86%)	63% (40%-79%)	1.47 (0.68-3.14)	0.32
ECOG performance status	0	74	13	99% (90%-100%)	88% (78%-94%)		Ref	
	1	37	12	95% (80%-99%)	86% (71%-94%)		1.90 (0.87-4.17)	0.11
	2	12	12	58% (27%-80%)	25% (6%-50%)		17.81 (7.55-41.98)	<0.001
	3	9	6	78% (36%-94%)	56% (20%-80%)		9.31 (3.33-26.09)	<0.001
p63	Negative	89	32	93% (85%-97%)	77% (66%-84%)	67% (55%-76%)	Ref	
	Positive	43	11	90% (76%-96%)	85% (70%-93%)	74% (57%-85%)	0.80 (0.40-1.58)	0.51
AJCC stage	I	25	6	96% (75%-99%)	88% (76%-100%)	83% (69%-100%)	Ref	
	II	81	17	96% (88%-99%)	88% (81%-96%)	79% (71%-89%)	0.96 (0.38-2.44)	0.93
	III	19	13	94% (67%-99%)	61% (42%-88%)	30% (14%-64%)	4.53 (1.71-12.01)	0.002
	IV	7	7	29% (4%-61%)			43.26 (12.34-151.64)	<0.001
Hormonal therapy	No	100	32	92% (84%-96%)	78% (68%-85%)	68% (57%-77%)	Ref	
	Yes	24	7	100% (NA)	96% (73%-99%)	77% (54%-90%)	0.86 (0.38-1.96)	0.72
Radiation therapy	No	53	16	87% (74%-93%)	77% (63%-86%)	70% (55%-81%)	Ref	
	Yes	74	25	97% (89%-99%)	81% (70%-89%)	69% (56%-78%)	1.01 (0.54-1.90)	0.97
Chemotherapy type	Anthracycline	68	18	97% (89%-99%)	85% (74%-92%)	77% (64%-85%)	Ref	
	Non-anthracycline	36	12	85% (67%-93%)	79% (61%-89%)	67% (46%-81%)	1.52 (0.73-3.15)	0.26
Surgery	Mastectomy	74	24	94% (86%-98%)	82% (71%-89%)	69% (56%-79%)	Ref	
	Lumpectomy	48	11	98% (85%-100%)	89% (75%-95%)	79% (63%-89%)	0.66 (0.32-1.34)	0.25
Histologic subtypes	Spindle cell	27	10	92% (73%-98%)	77% (56%-89%)	67% (44%-82%)	Ref	
	Squamous	40	15	90% (76%-96%)	77% (61%-88%)	64% (46%-77%)	0.97 (0.43-2.15)	0.94
	Mixed MBC/mesenchymal	54	15	94% (83%-98%)	80% (66%-89%)	73% (58%-83%)	0.71 (0.32-1.58)	0.40
	^#^Others	11	3	91% (51%-99%)	91% (51%-99%)	71% (34%-90%)	0.56 (0.15-2.05)	0.38
Estrogen receptor	No	111	36	92% (84%-9%)	77% (68%-84%)	67% (57%-75%)	Ref	
	Yes	21	7	95% (71%-99%)	90% (67%-98%)	75% (51%-89%)	0.95 (0.42-2.13)	0.89
Progesterone receptor	No	121	40	91% (85%-95%)	78% (69%-84%)	67% (57%-75%)	Ref	
	Yes	11	3	100% (NA)	100% (NA)	89% (43%-98%)	0.70 (0.22-2.26)	0.55
HER2-neu expression	No	119	39	92% (85%-96%)	80% (71%-86%)	68% (59%-76%)	Ref	
	Yes	13	4	92% (57%-99%)	77% (44%-92%)	69% (37%-87%)	0.87 (0.3-2.43)	0.79
Metastasis to the lung	No	102	18	96% (89%-98%)	88% (79%-93%)	81% (72%-88%)	Ref	
	Yes	30	25	80% (61%-90%)	53% (34%-69%)	27% (11%-44%)	7.85 (4.18-14.72)	<0.001
Metastasis to the bone	No	114	28	95% (88%-97%)	82% (74%-88%)	76% (67%-83%)	Ref	
	Yes	18	15	78% (51%-91%)	61% (35%-79%)	24% (7%-46%)	4.90 (2.57-9.34)	<0.001
Metastasis to the brain	No	123	34	92% (85%-95%)	83% (75%-89%)	72% (63%-80%)	Ref	
	Yes	9	9	100% (100%-100%)	33% (8%-62%)	22% (3%-51%)	5.53 (2.61-11.72)	<0.001
Metastasis to the liver	No	126	39	93% (86%-96%)	81% (73%-87%)		Ref	
	Yes	6	4	83% (27%-97%)	50% (11%-80%)		3.06 (1.08-8.67)	0.036

The potential predictors of outcome in univariable analysis were utilized for multivariable analysis. No difference in survival was observed in the early stages of MBC (stages I and II). Performance status proved to be predictive of OS (ECOG score 1: HR, 3.141 (95% CI, 1.00-9.88) (p=0.05); ECOG score 2: HR, 24.736 (95% CI, 1.92-318.73) (p=0.014)). Distant metastases to the lung (HR, 42.102 (95% CI 7.20-246.36) (p<0.001)) and brain (HR, 8.453 (95% CI, 1.88-38.04) (p= 0.005)) were statistically significant for worse OS in multivariable analysis (Table 5). Interestingly, patients with bone metastasis showed a tendency for a lower risk of death with an HR of 0.052 (95% CI, 0.01-0.48) (p=0.009). Other variables such as hormonal therapy, radiation therapy, and histologic subtype did not show any statistical significance for OS.

**Table 5:** Multivariable analysis for overall survival ECOG, Eastern Cooperative Oncology Group; HR, hazard ratio; CI, confidence interval; Ref, reference *Analysis was done using a reference level of no lung, bone, and brain metastasis.

Variable	HR (95% CI)	p-value
Stage II (ref = stage I)	0.671 (0.15-3.07)	0.61
Stage III (ref = stage I)	5.065 (1.02-25.27)	0.048
Stage IV (ref = stage I)	5.968 (0.55-64.47)	0.14
Hormonal therapy (ref = no)	0.142 (0.02-1.17)	0.07
Radiation therapy (ref = no)	3.626 (0.71-18.60)	0.12
ECOG 1 (ref = ECOG 0)	3.141 (1.00-9.88)	0.05
ECOG 2 (ref = ECOG 0)	24.736 (1.92-318.73)	0.014
ECOG 3 (ref = ECOG 0)	1.704 (0.18-16.48)	0.65
*Lung metastasis (ref = no)	42.102 (7.20-246.36)	<0.001
^*^Bone metastasis (ref = no)	0.052 (0.01-0.48)	0.009
^*^Brain metastasis (ref = no)	8.453 (1.88-38.04)	0.005

## Discussion

The identification of MBC has evolved over the past two decades since the official recognition of this subtype by the WHO [3]. Clinically, most MBC patients present with a well-circumscribed palpable mass, and the presentation resembles an invasive ductal carcinoma. However, previous studies reported larger tumor sizes, greater than 5 cm, which is associated with worse outcomes [4-6]. A large national database study by Pezzi et al. reported an increased proportion of MBC in older patients with a mean age of 61.1 years and a higher prevalence in African-American and Hispanic patients [6]. In our study cohort, the median age of diagnosis was 60 years (27-92 years), and most tumors were diagnosed at AJCC stage II, followed by stage I. The white population (76%) represented the dominant cohort of the study, which could possibly be due to the large referral center with better access to care and regional demographics of the MBC population.

Currently, MBC is classified into purely epithelial and mixed epithelial and mesenchymal components based on the updated fourth edition of the WHO classification for breast tumors [7]. It is a heterogeneous tumor with diverse histologic subtypes. Previous studies demonstrated variation in the prevalence of histologic subtypes in different ethnic populations. The spindle cell subtype resembles a low-grade sarcoma, which is the most common histologic subtype found in western patients and is reported to be associated with poor prognosis [8,9]. The squamous subtype was found to be more common in the Asian population [4,8]. However, Zhang et al. found the most common histology subtype of spindle cell carcinoma (34%) followed by the squamous subtype (31%) in the Chinese population [10]. In our study cohort, we identified the squamous subtype (29%) more commonly in our study population, followed by the spindle subtype (21%).

Tumor protein *p53* is a gene that functions as a tumor suppressor. The *p53* mutation has been commonly reported in MBC with a high frequency ranging from 53% to 64% [11,12]. Tumor protein *p63*, which is a member of the *p53* family, is expressed in MBC tumor cells and is also a myoepithelial marker [13]. Evidence suggests that *p63* can be utilized as a diagnostic marker for MBC but with no prognostic value [13,14]. We found 43 (31%) patients positive for *p63* expression in the entire cohort. No difference in outcome was found between patients positive for *p63* versus negative for *p63* expression.

MBC is known for its rapid tumor growth and an increased tendency for recurrence [3,15]. Typically, tumors are of higher nuclear grade and characterized by the absence of ER, PR, and HER2/neu expression [7,16,17]. Moreover, the prognosis of metaplastic triple-negative breast cancer is worse when compared to non-metaplastic triple-negative breast cancer [18]. In our study cohort, approximately two-thirds of the patients had metaplastic triple-negative breast cancer. The cohort with any hormone receptor positivity and treatment with hormonal therapy did not show any improvement in survival when compared with the metaplastic triple-negative cohort. We also observed the heightened potential of tumor metastasis to the lung (22%), followed by the bone (13%), brain (6%), and liver (4%). In addition, there was evidence of rare metastasis to the spleen and adrenal gland in the study cohort. Evidence suggests the metastatic spread of tumors to the lungs, bone, and brain via vasculature instead of lymphatics [19]. Patients with metastasis to the lung and brain in our study cohort demonstrated significantly poor survival outcomes.

Rakha et al. reported histologic subtype as an independent prognostic factor in MBC; the spindle subtype tumor had aggressive behavior with a worse prognosis as compared to the matrix-producing tumor and squamous subtype [8]. However, in other studies, the histologic subtype did not show any statistical significance as a prognostic factor [10,20]. In our study cohort, histologic subtypes did not prove to be a predictor of outcomes (Tables 6, 7). Nevertheless, young age (<40 years), skin invasion, and squamous carcinoma subtype with nodal involvement have been identified as independent predictors of outcome in MBC patients [15].

**Table 6:** Progression-free survival based on histology HR, hazard ratio; CI, confidence interval; Ref: reference; NA, not available; N, number of patients; MBC, metaplastic breast cancer #Other histology in our study includes ductal carcinoma in situ micropapillary pattern, heterologous type with matrix production, high-grade metaplastic carcinoma, large cells with intermingling mature plasma cells, matrix-producing carcinoma, and sarcomatoid features, and metaplastic carcinoma.

Variable	Variable level	N	Number of events	Estimated median (month)	One-year rate	Two-year rate	Five-year rate	HR (95% CI)	p-value
Histology	Spindle cell	27	11	74.8	92% (73%-98%)	73% (51%-86%)	56% (33%-74%)	Ref	
	Squamous	40	19	156.3	82% (67%-91%)	65% (48%-78%)	54% (37%-68%)	1.15 (0.55-2.42)	0.71
	Mixed MBC/mesenchymal	54	16	NA	88% (76%-95%)	76% (62%-86%)	66% (50%-78%)	0.69 (0.32-1.49)	0.34
	^#^Other	11	3	NA	91% (51%-99%)	82% (45%-95%)	72% (35%-90%)	0.54 (0.15-1.95)	0.35

**Table 7:** Overall survival based on histology HR, hazard ratio; CI, confidence interval; Ref: reference; NA, not available; N, number of patients; MBC, metaplastic breast cancer #Other histology in our study includes ductal carcinoma in situ micropapillary pattern, heterologous type with matrix production, high-grade metaplastic carcinoma, large cells with intermingling mature plasma cells, matrix-producing carcinoma, and sarcomatoid features, and metaplastic carcinoma.

Variable	Variable level	N	Number of events	Estimated median (month)	One-year rate	Two-year rate	Five-year rate	HR (95%CI)	p-value
Histology	Spindle cell	27	10	74.8	92% (73%-98%)	77% (56%-89%)	67% (44%-82%)	Ref	
	Squamous	40	15	NA	90% (76%-96%)	77% (61%-88%)	64% (46%-77%)	0.97 (0.43-2.15)	0.94
	Mixed MBC/mesenchymal	54	15	NA	94% (83%-98%)	80% (66%-89%)	73% (58%-83%)	0.71 (0.32-1.58)	0.40
	^#^Other	11	3	NA	91% (51%-99%)	91% (51%-99%)	71% (34%-90%)	0.56 (0.15-2.05)	0.38

In one of the large US population-based studies, 1,011 MBC patients were compared with 253,818 infiltrating ductal carcinoma (IDC) [21]. The study noted significantly worse five-year survival in MBC patients than in IDC patients (78% versus 93% (p<0.0001)). Besides, MBC was associated with higher tumor grades and larger tumor sizes. Similar findings were demonstrated in another study; authors reported decreased five-year OS when compared with invasive ductal carcinoma and triple-negative invasive ductal carcinoma (54.5% versus 85.1% versus 73.3% (p<0.001)) [5]. El Zein et al. also observed a worse prognosis in MBC than in triple-negative breast cancer (TNBC) [22]. The study documented that MBC patients had almost double the risk of local recurrence than TNBC patients (95% CI, 1.01-3.83 (p=0.05)). The authors also noted worse disease-free survival (DFS) and OS in MBC patients when compared with matched TNBC patients (p<0.001, p=0.033). Further review of the literature revealed a five-year OS ranging from 50% to 83% in various retrospective studies [10,17,19,23-29]. Most of the studies demonstrated a five-year DFS of 41%-65% [10,19,23,26-30], except for one study that demonstrated a five-year DFS of 84% [24].

In our study, we observed a five-year OS of 69% and PFS of 61%, which correlates with previous studies. On multivariable analysis, the stage of the disease, performance status (ECOG score), and distant metastasis to the lung, bone, brain, and liver proved to be significant predictors of outcomes in the MBC patient population. More importantly, on analysis for prognostication of MBC, we observed that patients with bone metastasis have a lower risk of death. On the other hand, a lower risk of progression was identified in the cohort of patients with liver metastasis. However, larger study samples would be needed to identify if these factors are truly associated with positive outcomes.

Our study is limited by its retrospective design with the possibility of selection bias and a small sample size. Few patients in the study cohort had missing variables; therefore, the total number of patients included for analysis for some of the specified variables was less than 136. We intended to do a descriptive analysis because of the above reasons. We utilized the pathology report documented by breast pathologists for MBC in our retrospective cohort study and did not review slides. Patients were not divided based on the type of presentation, such as primary first-time diagnosed MBC and concurrent malignancy in the contralateral breast. Another weakness of the study is that progression included both local and distant progression; no analysis was explicitly conducted for local recurrence. As far as treatment is concerned, we did not describe whether the patient received neoadjuvant or adjuvant chemotherapy, the combination of surgery with radiation therapy or hormonal therapy, or chemotherapy.

Despite the above limitations, the strength of the study is a sizable cohort of MBC patients over a long period of time depicting clinical and pathological characteristics.

## Conclusions

Our study demonstrated the aggressive nature of MBC with early progression and overall poor prognosis. Histologically, tumors were heterogeneous with no difference in outcome based on histologic subtypes. However, the stage of metaplastic breast cancer, poor functional status (ECOG score), and metastasis to the lung and brain are identified as independent predictors of poor survival outcomes in the entire cohort. Our experience with MBC with diverse clinicopathological findings and relevant prognostic factors is another addition to the literature. Tumor pathogenesis, diverse histology, and molecular heterogeneity pose a significant challenge in the diagnosis and management of MBC. The lack of a more specific therapeutic approach for this rare subtype of breast cancer is an unmet need that warrants further research and randomized clinical trials.
